# Crista Terminalis, Musculi Pectinati, and Taenia Sagittalis: Anatomical Observations and Applied Significance

**DOI:** 10.5402/2013/803853

**Published:** 2013-08-13

**Authors:** Abu Ubaida Siddiqui, Syed Rehan Hafiz Daimi, Kusum Rajendra Gandhi, Abu Talha Siddiqui, Soumitra Trivedi, Manisha B. Sinha, Mrithunjay Rathore

**Affiliations:** ^1^Department of Anatomy, All India Institute of Medical Sciences (AIIMS), Tatibandh, GE Road, Raipur, Chhattisgarh 492099, India; ^2^Department of Anatomy, Rural Medical College, Pravara Institute of Medical Sciences, Loni, Ahmednagar, Maharashtra 413736, India; ^3^Department of Cardiothoracic and Vascular Surgery, JJ Hospital of Grants' Medical College, Mumbai, Maharashtra 400008, India

## Abstract

*Background*. The complex architecture of the right atrium, crista terminalis (CT), and the musculi pectinati (MP) poses enormous challenges in electrophysiology and cardiac conduction. Few studies have been undertaken to substantiate the gross features of MP, in relation to the CT, but there is still scarcity of data regarding this. We tried to reinvestigate the gross arrangement of muscle bundles in the right atrium. *Methods*. Utilizing 151 human hearts and orientation of MP and its variations and relationship to the CT were investigated along with taenia sagittalis (TS). Patterns of MP were grouped in 6 categories and TS under three groups. *Result*. A plethora of variations were observed. Analysis of all the specimen revealed that 68 samples (45%) were of type 1 category and 27 (18%) fell into type 2 category. Prominent muscular columns were reported in 12 samples (8%). 83 samples (55%) presented with a single trunk of TS. Multiple trunks of TS were reported in 38 samples (25%). *Conclusion*. Samples with type 6 MP and type B/type C TS, which have a more complex arrangement of fibers, have a tendency to be damaged during cardiac catheterization. Nonetheless, the area as a whole is extremely significant considering the pragmatic application during various cardiac interventions.

## 1. Introduction

The crista terminalis (CT) is a well-defined fibromuscular ridge formed by the junction of the sinus venosus and primitive right atrium that extends along the posterolateral aspect of the right atrial wall [1]. It originates from the atrial septal wall medially, passes anterior to the orifice of the superior vena cava (SVC), descends posteriorly and laterally, and then turns anteriorly to skirt the right side of the orifice of the inferior vena cava (IVC).

It is an important anatomic landmark due to its close association with sinoatrial nodal artery and the origin of the musculi pectinati (MP, pectinate muscles). The MP are muscular ridges that extend anterolaterally from the CT to reach the auricle and may present in a number of variable forms and shapes/sizes. The largest and most prominent MP forming the bridge of the sulcus terminalis internally is called taenia sagittalis (TS), literally meaning *sagittal worm* [2].

 Occasionally, CT and the MP can be prominent, thus mimicking right atrial mass like a pseudomass, tumor, thrombus, or vegetation. An understanding of the anatomy and proper identification helps in avoiding misdiagnosis [1]. The MP with highly trabeculated muscle fibers predispose to arrhythmias. Moreover, prominent muscular columns with velamentous MP have implications for cardiac catheterization.

In common atrial flutter, CT has been described as a natural barrier to the cardiac conduction system [2]. Studies using intrathoracic echocardiography have shown that two-thirds of focal right atrial tachycardia, seen in the absence of structural heart disease, arises along the CT. Ablation targeting the CT has also been used in patients with inappropriate sinus tachycardia [3]. CT is the most obvious muscle, and the wall of the right atrium is not of uniform thickness because of the variable patterns of the MP and TS [4]. 

The CT of His is a significant structure in several forms of atrial tachyarrhythmia and occasionally is a target for radiofrequency catheter procedures [5]. It may act as an important anatomic landmark for such arrhythmias by initiating ectopic atrial beats [6]. Occurrence of intercaval conduction block during atrial flutter seems to indicate either an anatomic or an electrophysiological predisposition for conduction abnormalities. Experimental and clinical studies suggest that CT forms the anatomic substrate underlying intercaval conduction block. The CT may provide the substrate for a central obstacle in a macroreentrant circuit. This may explain the occasional occurrence of typical atrial flutter in healthy individuals [7].

 Even though a few studies have been undertaken to analyze the gross features of MP in relation to the CT [2], there is still scarcity of data from this part of the world. Taking into consideration the previous stated facts, this study was planned and executed to describe, analyze, and chart the morphologic patterns of the MP and TS. In concurrence to some earlier studies, we have tried to emphasize upon the structural variants of the MP and the TS (if present) [2]. Moreover, the study has prospects related to the development of thrombus formation and possible implications to identify sites of iatrogenic cardiac injury pertaining to catheterization [2].

## 2. Material and Methods

The study was approved by the Institutional Ethical Committee of our university. The purpose of this study was to reinvestigate the gross arrangement of the principal muscle bundles in the right atrial wall. For the study, we utilized 151 human hearts acquired from the cadavers received in the Department of Anatomy of the institute, which is located in the Western part of Maharashtra state of India. Out of these, 150 were formalin fixed samples, preserved in our departmental collection, and one was from a fresh body. The specimen selected for the study did not show any evidence of previous surgical interventions and were free from traumatic injuries as well as any sort of evident and observable pathology. To tide over the examiner variability, each specimen was examined independently by 2 coauthors. All the photographs were taken by the authors using an 8 megapixel Sony Cybershot digital camera and edited on Adobe Photoshop version 7 software.

 To achieve a trustworthy visualization of the CT and MP, the superior vena cava (SVC) and inferior vena cava (IVC) were cut open posteriorly along a linear axis, thus exposing the right atrium. A second incision was made perpendicular to the original cut, parallel to the coronary sulcus. This was done to preserve the near normal morphology of the CT and the MP.

 We have mainly targeted the orientation of the MP, their variations, and their relationship to the CT. Moreover, the interesting features of the TS were also taken into account and analyzed.

## 3. Results 

The CT was present in all of the 151 specimens examined. Moreover, a plethora of variations, as regards the morphological patterns concerned with the MP and TS, was observed and recorded. The basis for the classification may be attributed to the developmental pattern of the pectinate muscle as well as the right atrial conduction physiology. To enhance a methodical analysis amongst the specimens undertaken for the study and to facilitate a proper understanding of the variable patterns, the results were classified as under the following.

*(A) Observations on MP.* Taking into account the course and variable pattern of MP—The architecture of the MP was classified into six various patterns.

**Figure 1:**
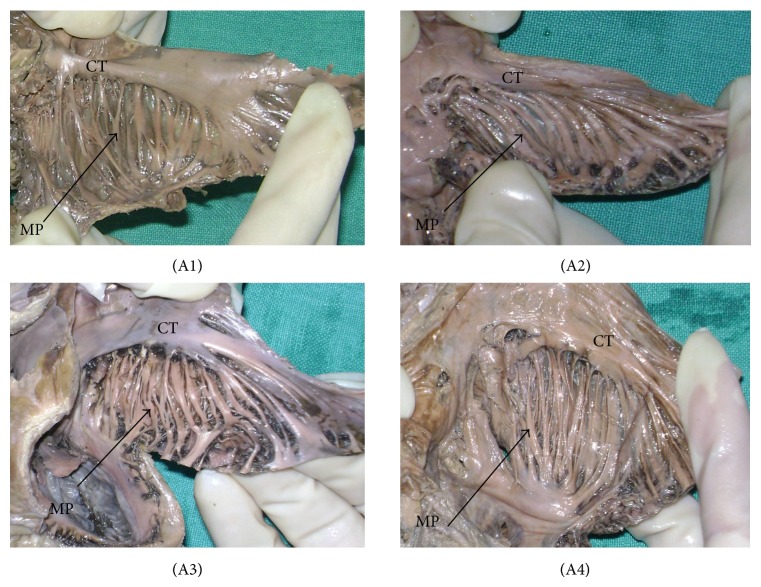
The patterns of musculi pectinati (MP). The black arrow indicates MP. CT = crista terminalis. (A1) type 1, (A2) type 2, (A3) type 3, (A4) type 4.

**Figure 2:**
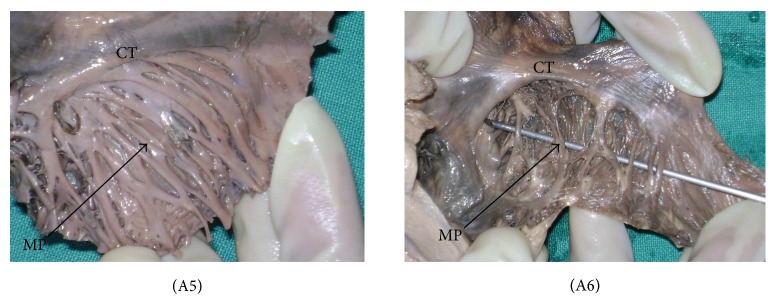
The patterns of musculi pectinati (MP). The black arrow indicates MP. CT = crista terminalis. (A5) type 5, (A6) type 6.

1. Type 1—MP oriented perpendicular to the CT with equidistant spacing—shown as (A1) (Figure 1).
2. Type 2—MP oriented parallel to the CT with equidistant spacing—shown as (A2) (Figure 1).
3. Type 3—combination of type 1 and type 2, with more than one common muscular trunk—shown as (A3) (Figure 1).
4. Type 4—branching of the MP—shown as (A4) (Figure 1).
5. Type 5—interlacing trabeculations, nonuniform MP organized in a haphazard, and trabecular pattern with numerous crossovers—shown as (A5) (Figure 2).
6. Type 6—prominent muscular columns of MP—shown as (A6) (Figure 2).

Analysis of all the specimens revealed that 68 samples (45%) were of type 1 and 27 (18%) fell into type 2 category. Prominent muscular columns (type 6) were reported in 12 samples (8%). The results are summarized in Table 1 and photographically represented in Figures 1 and 2.

*(B) Observations on TS.* The TS was classified into three groups.

**Figure 3:**
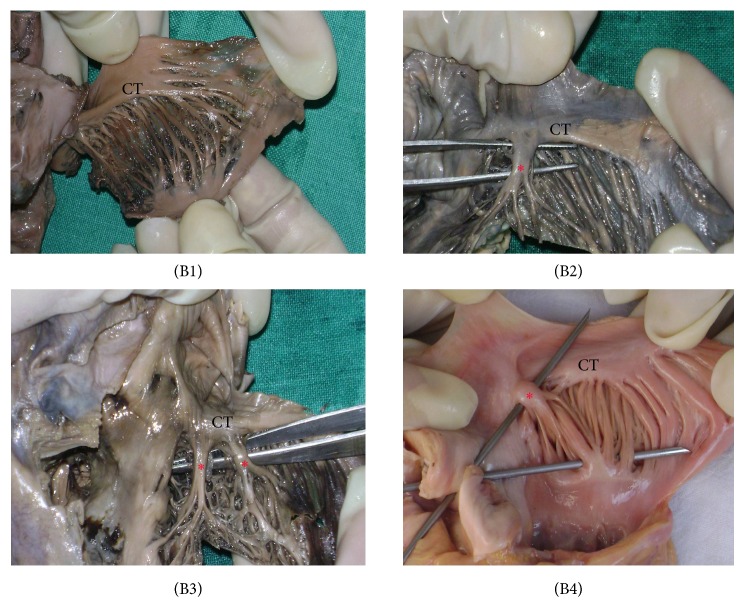
The patterns of taenia sagittalis (TS). CT = crista terminalis. (B1) type A, (B2) type B, and (B3)/(B4) type C. The red asterix in (B2), (B3), and (B4) indicates the prominent TS. Please note that specimen (B4) is from a fresh cadaver.

1. Type A—TS absent—shown as (B1) (Figure 3).
2. Type B—single trunk of TS—shown as (B2) (Figure 3).
3. Type C—multiple trunks of TS—shown as (B3), (B4) (Figure 3).

83 samples (55%) presented with a single trunk of TS. An interesting observation was regarding the presence of multiple trunks of TS in 38 samples (25%). The results are summarized in Table 2 and photographically represented in Figure 3.

## 4. Discussion

The complexity of atrial geometry dictates that the spread of activation from the site of origin of cardiac impulse shall be restrained by naturally occurring barriers [3]. Investigating the mechanisms underlying the genesis and conduction of electrical excitation in the atria at physiological and pathological states is of great importance.

Recent studies have determined the role of the crista terminalis in the mechanisms of cardiac arrhythmias (atrial flutter/atrial fibrillation). It is also intriguing to speculate that structural abnormalities of the CT and MP may be the primary abnormality in patients with atrial flutter and may explain the occurrence of atrial flutter even in patients with grossly normal atria [8–10]. In a study conducted on twenty-three patients, it was demonstrated that approximately two-thirds of focal right atrial tachycardia occurring in the absence of structural heart disease arose along the CT [2, 3, 11]. 

Utilizing the CT and MP tissues of 10 adult dogs, it was proposed that the crista terminalis (CT) is known to initiate and maintain atrial arrhythmia and is affected by autonomic tone, but the underlying mechanisms were poorly understood [12]. Earlier workers have hypothesized that the development of atrial flutter can be attributed to the geometry of CT and the associated structures [13]. 

Despite the extensive literature concerning atrial arrhythmias, there are relatively few papers on the anatomy of the atrial chambers. The right and left atria are characterized by morphologically distinct appendages. The right atrium contains prominent muscular bundles and an extensive array of pectinate muscles. The distal ramifications of the CT lead to the “flutter” isthmus. By contrast, the left atrium has relatively smooth walls. The structure of the atrium is much more than an anatomic curiosity. It has practical implications for mapping and interventional procedures [14].

Recent electrophysiological observations have suggested that the right atrial posterolateral wall, which contains the CT and sinus venosus, may be an arrhythmogenic substrate such as micro- or macroreentrant right atrial arrhythmia. CT and its vicinity are considered to be the focus of atrial ectopic beats or sinoatrial reentry and the posterior boundary of flutter. Although this phenomenon has been attributed to anisotropy, the anatomy of this area of the atrium has not been described in detail [15]. It was also hypothesized that the limited transverse conduction capabilities of the CT may contribute to the development of atrial flutter [16–18]. Apart from being the causative factor for the arrhythmias, a prominent CT is a variant of normal heart anatomy that may mimic right atrial mass-like tumor, thrombus, or vegetation [1, 19].

Atrial dysfunction is the most common pathology in the heart, which may also accompany other severe cardiac diseases, for example, congestive heart failure. A better understanding of fundamental mechanisms underlying atrial function and dysfunction is of great benefit to therapeutic approaches. Anatomical structure, interatrial coupling, fast conduction bundles, and electrophysiological heterogeneity seem to play an important role for atrial excitation conduction during physiological and pathological conditions. The most important anatomical structures for initiation and conduction of atrial excitation are the sinoatrial node, the CT, MP, and the interatrial connections. Understanding atrial electromechanical function requires knowledge of anatomy, electrophysiology, and excitation conduction, as well as active and passive mechanics. The detailed model of human atria provides a useful and complementary tool to investigate the dynamical behaviour of atria [20].

It has been postulated that large MP ridges provide a natural substrate for the initiation of intra-atrial reentry and prolong the life spans of reentrant wave fronts, thus determining “flutter-like” or “fibrillation-like” activity, respectively, in isolated canine atrial tissue [21].

As early as 1909, Flack emphasized the importance of MP in atrial contraction and argued that the function of MP of atrium has been neglected. In 1920, Papez described and established more fully the arrangement of the atrial muscles of mammalian hearts, confirming the important role of MP. CT is the most obvious muscle, and the wall of the right atrium is not of uniform thickness because of the variable patterns of the MP and TS [4].

It would not be an exaggeration to state that the orientation of fibres from the CT and MP forms an anatomic/electrophysiological basis for intercaval conduction block. MP with highly trabeculated muscle fibres may facilitate the nonuniform spread of the excitatory impulse. Due to this arrangement of muscle bundles, the patient is predisposed to severe atrial arrhythmias. More practically, one of the most common ways of treating atrial flutter is the use of radiofrequency catheter ablation in which the tissue suspected of causing arrhythmias is ablated. It is during this ablation procedure that the morphology of the MP is pertinent as it carries a risk of iatrogenic myocardial injury (especially in (A6), Figure 2 and (B2), (B3), and (B4), Figure 3). Nonetheless, these hearts are at an enhanced risk of being damaged during catheterization, as during the procedure, the catheter tip has chances of being stuck deep to the prominently disposed muscular columns (MP)—which may further result in perforation of the atrial wall or the associated muscle bundles. The present study enlightens the variational morphology of the MP and the prominent TS. The proposed classification and nomenclature for the various arrangements of fibres should prove helpful in determining the geometry of these structures relevant to cardiac physiology and cardiac interventions. 

Our study was carried out to categorize the MP and the TS so that further prospective studies can be taken up and a uniform method of classification, while undertaking clinical procedures, can be planned and executed. Moreover, clinical studies related to the structures, especially in patients of arrhythmias, should be taken up by the cardiologists and cardiac fraternity to establish a valid tool for future classification and nomenclature of these hitherto poorly unexplored anatomic structures (mainly MP and TS).

In view of the scanty literature pertaining to the MP and TS, this paper has tried to investigate the gross morphological arrangement of the principal muscular bundles. This may prove beneficial for the interventional operator to have beforehand anatomically precise information. 

## 5. Conclusion

Morphology of CT and MP has a pragmatic role in electrophysiology and cardiac conduction, with inherent implications in arrhythmias. CT is a significant anatomic landmark with clinical relevance to interventional catheterization procedures. By the virtue of our study, it is proposed that hearts with type 6 MP and type B/type C TS, which have a more complex arrangement of fibres, have a tendency to be damaged during cardiac catheterization. While performing a catheterization, it is possible that the tip of the cardiac catheter may be stuck behind the TS or any of the MP branches. A further injudicious push at this point could result in right atrial perforation. Irrespective of the types of MP and the TS, the area as a whole is extremely significant in order to avoid trauma to the SA nodal artery, thrombosis of the SA nodal artery, and trauma to the SA node.

## Figures and Tables

**Table 1:** Patterns for MP (*n* = 151).

	Type 1	Type 2	Type 3	Type 4	Type 5	Type 6
Pattern observed	Perpendicular to CT	Parallel to CT	Combination of type 1 and type 2	Branching	Interlacing trabeculations	Prominent muscular columns
Number of heart samples	68	27	16	14	14	12
Percentage (%)	45	18	11	9	9	8
Photo/figure	(A1) (Figure 1)	(A2) (Figure 1)	(A3) (Figure 1)	(A4) (Figure 1)	(A5) (Figure 2)	(A6) (Figure 2)

**Table 2:** Patterns for TS (*n* = 151).

	Type A	Type B	Type C
Pattern observed	No TS	Single TS	Multiple TSs (more than one)
Number of heart samples	30	83	38
Percentage (%)	20	55	25
Photo/figure	(B1) (Figure 3)	(B2) (Figure 3)	(B3)/(B4) (Figure 3)
